# Early prediction of in-hospital mortality utilizing multivariate predictive modelling of electronic medical records and socio-determinants of health of the first day of hospitalization

**DOI:** 10.1186/s12911-023-02356-4

**Published:** 2023-11-13

**Authors:** Daniel Stoessel, Rui Fa, Svetlana Artemova, Ursula von Schenck, Hadiseh Nowparast Rostami, Pierre-Ephrem Madiot, Caroline Landelle, Fréderic Olive, Alison Foote, Alexandre Moreau-Gaudry, Jean-Luc Bosson

**Affiliations:** 1 Life Science Analytics, Clinical Solutions, Elsevier, Berlin, Germany; 2 Elsevier Health Analytics, London, UK; 3 Public Health Department, CHU Grenoble Alpes, Grenoble, F-38000 France; 4 Digital Services Management, CHU Grenoble Alpes, Grenoble, F-38000 France; 5 TIMC CNRS UMR5525, Université Grenoble Alpes, Grenoble, F-38000 France

**Keywords:** All-cause in-hospital mortality, Data at hospital admission, Machine learning, Clinical decision making

## Abstract

**Background:**

In France an average of 4% of hospitalized patients die during their hospital stay. To aid medical decision making and the attribution of resources, within a few days of admission the identification of patients at high risk of dying in hospital is essential.

**Methods:**

We used de-identified routine patient data available in the first 2 days of hospitalization in a French University Hospital (between 2016 and 2018) to build models predicting in-hospital mortality (at ≥ 2 and ≤ 30 days after admission). We tested nine different machine learning algorithms with repeated 10-fold cross-validation. Models were trained with 283 variables including age, sex, socio-determinants of health, laboratory test results, procedures (Classification of Medical Acts), medications (Anatomical Therapeutic Chemical code), hospital department/unit and home address (urban, rural etc.). The models were evaluated using various performance metrics. The dataset contained 123,729 admissions, of which the outcome for 3542 was all-cause in-hospital mortality and 120,187 admissions (no death reported within 30 days) were controls.

**Results:**

The support vector machine, logistic regression and Xgboost algorithms demonstrated high discrimination with a balanced accuracy of 0.81 (95%CI 0.80–0.82), 0.82 (95%CI 0.80–0.83) and 0.83 (95%CI 0.80–0.83) and AUC of 0.90 (95%CI 0.88–0.91), 0.90 (95%CI 0.89–0.91) and 0.90 (95%CI 0.89–0.91) respectively. The most predictive variables for in-hospital mortality in all three models were older age (greater risk), and admission with a confirmed appointment (reduced risk).

**Conclusion:**

We propose three highly discriminating machine-learning models that could improve clinical and organizational decision making for adult patients at hospital admission.

**Supplementary Information:**

The online version contains supplementary material available at 10.1186/s12911-023-02356-4.

## Background

In France, approximately 4% of all patients admitted to a hospital die during their in-patient hospital stay (according to the 2022 database of the Technical Agency for Information on Hospital Care (ATIH) [1]). The early detection of patients with a high risk of dying in hospital may improve organizational and clinical decision making and help to determine the scale of required medical resources [2]. However, most established mortality prediction systems such as the SAPS score [3–5], SOFA score [6], and APACHE score [7–9] focus on adult intensive care unit admissions, and the consideration of cases during their entire hospitalization (including patients who are not admitted to intensive care) is less frequent [10]. Machine learning algorithms offer the advantage of providing a predictive tool with high flexibility based on a large set of information from electronic health records (EHR) [2, 11–13].

In recent years, the number of medical studies utilizing different kinds of machine learning algorithms for clinical decision support has increased [14, 15]. Machine learning algorithms may improve the proper early identification of patients at risk of in-hospital mortality. Several studies have applied machine learning algorithms to predict in-hospital mortality of adult patients, identifying several risk factors among vital signs and laboratory tests [10, 11, 16–19]. In addition, machine learning methods have been used to predict in-hospital mortality from sepsis [20] and diabetic patients in intensive care units [21].

Overall, studies based on a general and entire hospital population are rare. The predictive value of matched administrative data and socio-economic variables needs to be investigated.

## Methods

### Aim and design

Our primary goal was to build and compare multiple machine learning models that predict in-hospital mortality for adult patients using the diverse data available at the beginning of their hospital stay. The secondary goal was to use the best models to identify the most important risk factors of in-hospital mortality.

### Study population

This study is based on 273,696 admissions to Grenoble Alpes University Hospital, France (CHUGA) between January 1, 2016 and December 31, 2018 [22]. Briefly, only adult patients (aged ≥ 18 at admission) with a length of stay of more than two days were included. Patients with geriatric long stays or permanent hospitalization, day clinic patients, or those with coding errors were excluded. Patients who died between the second and 30th day (included) after admission were considered as the case group. Patients who did not die in this time frame were considered as the control group. The dataset was randomly divided into a training/validation set (80%) and a benchmark set (20%).

### Database

The de-identified medical and administrative data from the Clinical Data Warehouse (CDW) PREDIMED [23] includes administrative and demographic information, hospitalization details, laboratory results, diagnoses, procedures, and medications.

Fifty-one distinct social determinants of health (SDOH) from the French national institute for statistical and economic studies (INSEE) corresponding to every patient´s home address were added. Patients´ home addresses had been geocoded using the National Address Database geocoding service. In detail, six age-group related, 10 household–related, nine population-structure related, 20 housing-related, and seven activity-related variables available for inhabitants of the Grenoble area were included in this analysis (detailed list in the Supplementary Material: Table S1).

### Missing data

To reflect the specific circumstances and clinical considerations during data collection and avoid introducing potential biases through imputation, missing values for categorical and continuous variables were labelled as zero.

### Variable selection and correlation analysis

A total of 11 different categories of variables were considered for modelling, including age at admission, sex, mode of admission, hospital department/unit code, home address postal code type (urban, rural, semi-rural, or none), primary discharge diagnosis from any previous hospital stay, medication score (defined as the number of different drugs prescribed during the first day after admission [24]), laboratory tests (ordered and high, low, or normal result), Classification of Medical Acts (CMA) code(s), Anatomical Therapeutic Chemical (ATC) medication code(s), and SDOH (Table 1). To limit variables to a manageable number CMA and ATC codes were truncated to the first 3 and 4 characters respectively.

**Table 1:** Variable selection and criteria

Variable category	Comment data type	Time frame	Selection
Age at admission	continuous	at admission	/
Sex	categorical	at admission	/
Mode of admission	categorical	at admission	*
Hospital department—unit (ward) code	first 4 digits, categorical	max one day after admission	*
Postal code type (urban, rural, semi-rural, or none)	categorical	at admission	*
Discharge diagnosis from previous hospital stay	first 3 characters, categorical	previous hospital stay	*
Medication score (number of medications given)	continuous	max one day after admission	/
Laboratory test (tested, high, low, normal)	categorical	max one day after admission	**
Procedural codes—CMA	first 4 characters, categorical	max one day after admission	*
Medication—ATC codes	first 3 digits, categorical	max one day after admission	*
Socio-economic variables^a^	continuous	at admission	/

^a^Table S1

^*^Chi-square test (*p*-value < 0.05), present in at least 5% of all admissions per group

^**^Fisher-exact test (*p*-value < 0.05), present in at least 5% of all admissions per group for every laboratory test and the sub-categories (high, normal, low). CMA (Classification of Medical Acts code), ATC (Anatomical Therapeutic Chemical code)

The number of variables were preselected to reduce model complexity and to avoid overfitting. Categorical variables were selected using chi-square testing [25]. In addition, only variables available for at least 5% of admissions per group (case/control) or absent in one group but present in at least 5% of admissions in the other group were retained. A laboratory test was only retained if there was a significant difference between cases and controls in orders for the test and if the test results (high, normal, low) were significantly (Fisher exact test) different between cases and controls. For these tests a *p*-value < 0.05 was considered statistically significant. These multi-level categorical variables were introduced into the model as binary dummy variables. In a final step of dimension reduction, variables were tested for interdependency using Pearson correlation analysis [26]. Categorical and continuous variables with a correlation coefficient > 0.9 are presented by only one of the correlated variables. Correlation between categorical and continuous variables was tested using point-biserial correlation [27]. Finally, continuous variables such as age at admission, medication score, and socio-economic variables were scaled using the scikit-learn min–max scaler [28] to express each variable in a range between 0 and 1. A descriptive analysis of all primary and secondary diagnoses (3-digit ICD10 code) during the entire hospital stay (for both cases and controls) was done. It should be noted that final diagnoses for the current hospital stay could not be used as a variable for predictive modelling since the time stamps of these entries in the electronic medical record were not accurate enough. We selected diagnoses with a false discovery rate (FDR) of < 0.01 using chi-square testing [25] and the Benjamini–Hochberg correction for multiple testing [29]. Variables are described by numbers and percentages, means and their 95% confidence interval (95% CI) and standard deviations. Results are presented as Odds Ratio (OR) and their 95% CI. All statistical tests were two-tailed.

### Machine learning

**Fig. 1:**
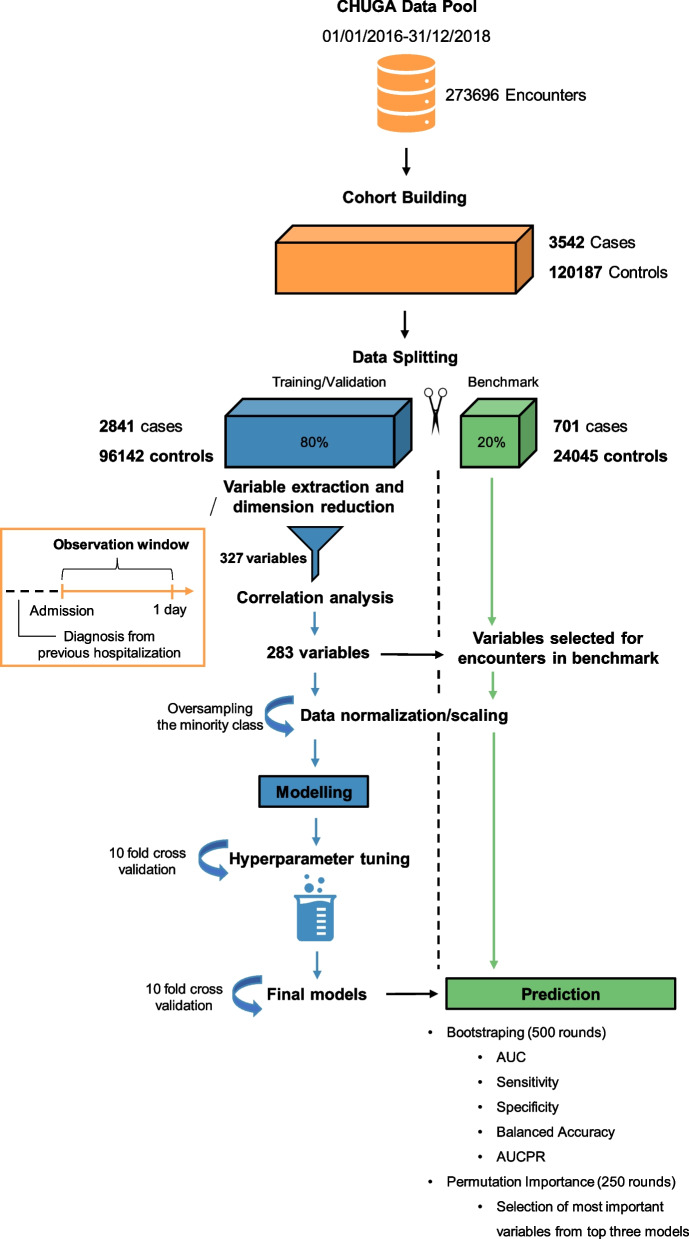
Workflow to detect early clinical factors associated with in-hospital mortality. AUC: area under the curve; AUCPR: area under the precision recall curve

To build predictive models and identify potential risk factors for in-hospital mortality, various supervised machine learning algorithms were investigated. This set of algorithms covers a wide range of different model classes, such as regression algorithms e.g., Logistic Regression (LR) [30], and instance-based algorithms e.g., Support Vector Machines (SVM) [31]. In addition, Bayesian algorithms e.g., Naive Bayes (NB) [32], ensemble algorithms e.g., Random Forest (RF) [33], Xgboost [34] and light gradient boosting machine (LightGBM) [35], deep learning algorithms such as Multilayer Perceptrons (MLP) [36], and the non-parametric algorithm k-nearest neighbors (KNN) [37] were used. Moreover, the scikit-learn dummy classifier (DC) [28], which makes predictions regardless of the input variables, was included as a benchmark model serving as a baseline reference to evaluate the performance of more sophisticated machine learning models. For each model, if applicable, algorithm hyperparameters were optimized using distributed asynchronous optimization [38] (Table S2(a)). For LightGBM and Xgboost, ten rounds of early stopping where applied, using the balanced accuracy and logistic loss as an evaluation metric, respectively. To improve model generalization and performance of the strongly unbalanced data set, the minority group (cases) was oversampled in the training data set at a ratio of one to one using the RandomOverSampler function from the imbalanced-learn library [39]. In each model, using the random search method, the algorithm hyperparameters were optimized based on the F1-score evaluation metric [40, 41], which is the harmonic mean between precision and recall (also known as prediction sensitivity). In addition, a ten-fold cross validation was applied to derive a more reliable model evaluation. The final models were tested and used for predicting in-hospital mortality, using the “benchmark” dataset (Fig. 1).

Model performances were evaluated by assessing prediction sensitivity, specificity, balanced accuracy (formulas 1, 2, 3, 4 and 5); area under the curve (AUC), and area under the precision recall curve (AUCPR); 95% confidence intervals (CI) were determined using a 500-step bootstrap analysis on the benchmark dataset.

Bootstrapping is a resampling technique that involves randomly sampling with replacement from the original dataset to create multiple new datasets. By repeatedly fitting the model to these resampled datasets, we estimated the variability and uncertainty of the model performance metrics by calculating the 2.5th percentile and the 97.5th percentile of the distribution of the performance metric after bootstrapping.

1 $$Sensitivity\;(Recall)=\frac{TP}{TP+FN}$$

2 $$Specificity=\frac{TN}{TN+FP}$$

3 $$F1\;Score=2\times\frac{Sensitivity\times Specificity}{Sensitivity+Specificity}$$

4 $$Balanced\;Accuracy=\frac{Sensitivity+Specificity}2$$

5 $$Precision= \frac{TP}{TP+FP}$$

*The number of true positives (TP), the number of false positives (FP), the number of false negatives (FN) and the number of true negatives (TN).*

The top three models were selected based on their balanced accuracy. In addition, confusion matrices, receiver operating characteristic (ROC) curves and precision-recall curves were generated. Variable importance in these final models was determined using 250 rounds of permutation importance calculation using the Python scikit-learn [28] permutation importance function, where the balanced accuracy was used as an evaluation metric. To determine the least number of variables needed to achieve similar model performances (balanced accuracies) in comparison to the full set of variables. For the top three models, the top 2–150 variables were re-modelled and plotted regarding their resulting balanced accuracy. The least necessary number of variables was determined by identifying the point on the resulting curve where the slope flattened. For each of the top three models these variables were finally plotted after sorting them by their corresponding mean balanced accuracy and the most influential variables were determined by identifying the point on the curve where the slope flattens.

Decision Curve Analysis (DCA) [42] was performed for the top three performing models in our study using the full set of variables. DCA is a useful tool for assessing the clinical utility of predictive models, evaluating their performance across different threshold probabilities. The prevalence was preset to 4.5%, representing the approximate prevalence observed in our dataset.

## Results

### Study population

**Fig. 2:**
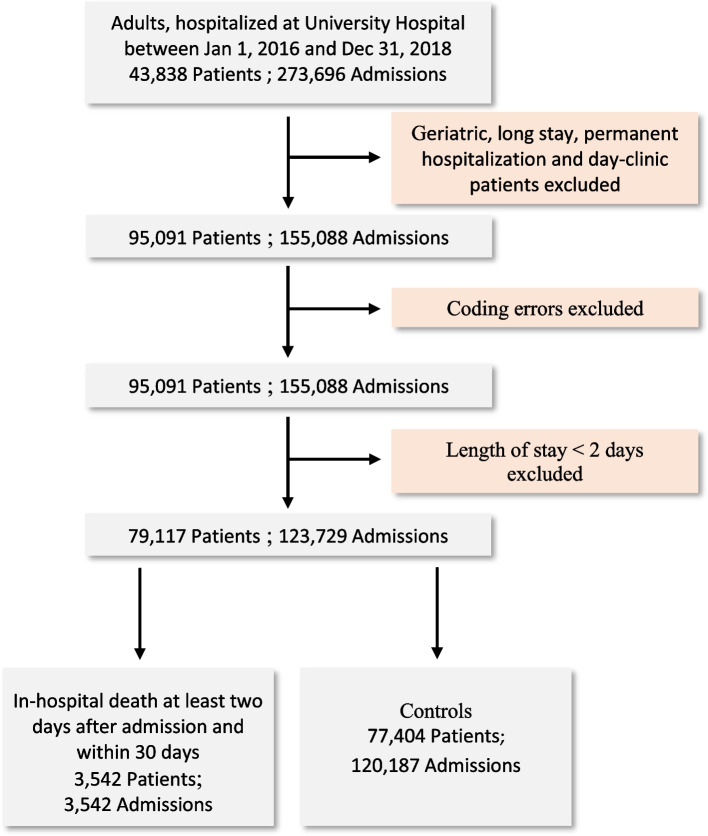
Flowchart for dataset selection

Among 123,729 admissions in the selected dataset [22], 3542 (2.86%) of admissions in 79,117 (4.48%) eligible patients were considered as cases (in-hospital death) and 120,187 admissions were used for the control group (Fig. 2).

### Variables

The variables retained included seven modes of admission, seven hospital departments, three different diagnoses from previous hospital stays, 51 laboratory tests, 20 procedures, 29 different medications, two postal code types and 51 different social determinants of health (Tables S1 and S3 (a). Completeness was high: 100% for age, sex, mode of admission, hospital department and SDOH, 99.97% for postal code type, 87.14% for laboratory tests, 84.37 for CMA procedural codes, and 92.34% for ATC medication codes; with an exception of 27.93% for the discharge diagnosis from a previous hospital stay.

Our correlation analysis identified six highly correlated (Pearson correlation coefficient > 0.9) continuous variables of which three were retained (all SDOH) and 49 highly correlated (Pearson correlation coefficient > 0.9) categorical variables (all laboratory tests categorized as “tested”) of which eight were retained (Table S4 (a) and (b)). Finally, a total of 283 variables were used for predictive modelling. We could not detect any correlation between the remaining categorical and continuous variables (point-biserial correlation coefficient ≤ 0.9).

### Analysis of the study population

Male patients were significantly (*p*-value < 0.05) more frequent in the case group compared to the control group (2036 (57.8%) compared to 60,120 (50.0%) respectively). Patients in the case group were significantly (*p*-value < 0.05) older than controls (mean age 75.9 [95% CI 75.4 -76.4] vs. 60.5 [95% CI 60.4–60.6]. Moreover, the number of medications prescribed in the observation window was significantly higher (*p*-value < 0.05) in the case group compared to the control group (Table S3 (b)).

Differences between cases and controls were identified for six primary and 85 secondary diagnoses (FDR < 0.01) (Table S5 (a) and (b)). We observed a significantly higher proportion of patients in the case group with a primary diagnosis of cardiogenic shock (OR = 12.51; 95% CI 10.94–14.32), malignant neoplasm of main bronchus (OR = 12.08, 95% CI 10.32–14.14), acute respiratory failure (OR = 9.09, 95% CI 7.90–10.38), heart failure (OR = 3.45, 95% CI 3.06–3.90), antineoplastic radiation therapy (OR = 3.09, 95% CI 2.73–3.49) and stroke due to thrombosis of precerebral arteries (OR = 2.90, 95% CI 2.49–3.37) compared to the control group.

### Predictive modelling

After randomly splitting the data, with 2,841 cases and 96,142 controls assigned to the training/validation group; and 701 cases and 24,045 controls to be used as the benchmark dataset.

**Fig. 3:**
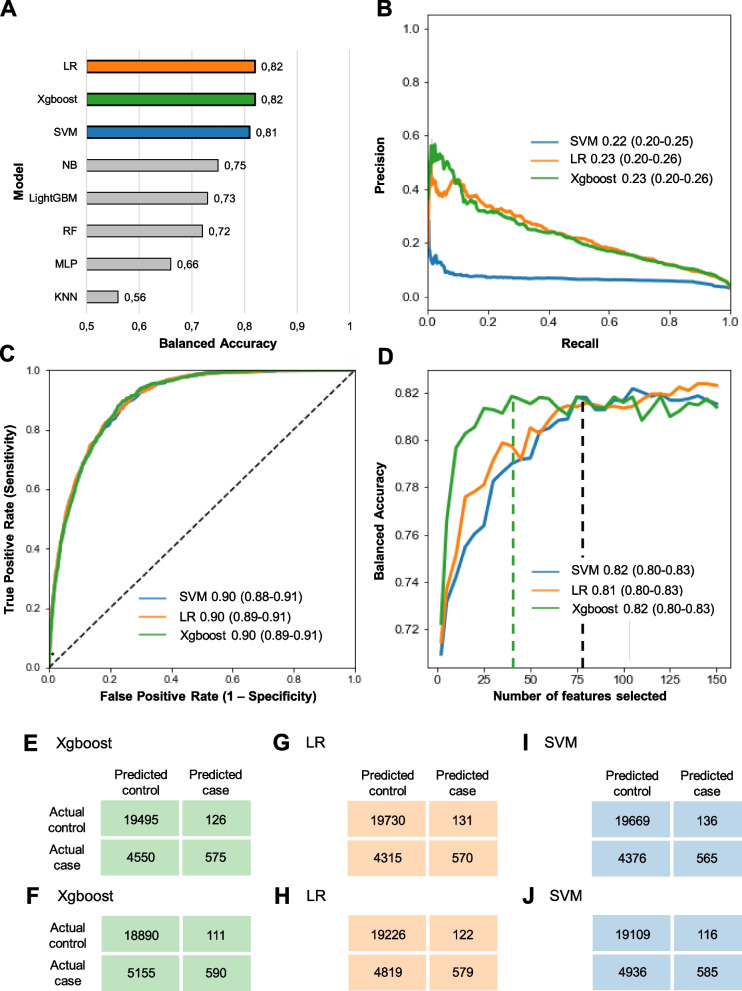
Model performances on the unseen benchmark dataset. **A** Balanced accuracy for all machine learning algorithms. **B** Precision-Recall curve based on all variables and the corresponding area under the precision recall curve (AUCPR). **C** Receiver operating characteristics (ROC) curve based on all variables and the corresponding area under the curve (AUC). **D** Balanced accuracy, based on the number of most important features selected, the dashed grey line illustrates the selected threshold for LR and SVM, dashed green line for Xgboost. Confusion matrices for **E** Xgboost based on all variables, **F** Xgboost based on top 45 variables, **G** LR based on all variables, **H** LR with the top 75 important variables, **I** SVM based on all variables and **J** SVM based on top 75 most important variables. Numbers in brackets in (**B**-**D**) correspond to the 95% confidence intervals determined by 500 bootstrappings. Logistic regression (LR), support vector machine (SVM), naive bayes (NB), light gradient boosting machine (LightGBM), multilayer perceptron (MLP), k-nearest neighbors (KNN) and random forest (RF)

The extracted training/validation dataset was used to optimize the corresponding algorithm hyperparameters (Table S2 (b)) and the final model was tested using the unseen benchmark dataset (Table 2, Fig. 3). Based on the balanced accuracy of the benchmark dataset, SVM and LR showed the best performances with a balanced accuracy of 0.81 (0.80–0.82) and 0.82 (0.80–0.83) respectively, when all variables were used for modelling. To reduce model complexity, we set out to determine the least number of variables necessary to achieve similar model performances (balanced accuracy) for SVM, LR and Xgboost in comparison to the full variable list. By selecting the top 2–150 variables based on the variable importance score, we determined that at least 75 of the most impactful variables were necessary to achieve a balanced accuracy of 0.82 (95% CI 0.80–0.83) for SVM and 0.81 (95% CI 0.80–0.83) for LR respectively. For Xgboost, at least 45 of the most impactful variables were necessary to achieve a balanced accuracy of 0.82 (95% CI 0.80–0.83) (Fig. 3D).

**Table 2:** Metrics for all classification models. 95% confidence intervals (95% CI) derived from 500 bootstraps by using all variables. Selected models are highlighted in bold

Model	AUC (95% CI)	AUCPR (95% CI)	Sensitivity (95% CI)	Specificity (95% CI)	Balanced accuracy (95% CI)	F1 Score (95% CI)
**LR**	0.90 (0.89–0.91)	0.23 (0.20–0.26)	0.81 (0.78–0.84)	0.82 (0.82–0.83)	0.82 (0.80–0.83)	0.55 (0.54–0.56)
**Xgboost**	0.90 (0.89–0.91)	0.23 (0.20–0.26)	0.82 (0.79–0.85)	0.81 (0.81–0.82)	0.82 (0.80–0.83)	0.55 (0.54–0.57)
**SVM**	0.90 (0.88–0.91)	0.22 (0.20–0.25)	0.81 (0.78–0.83)	0.82 (0.81–0.82)	0.81 (0.80–0.82)	0.55 (0.54–0.57)
NB	0.80 (0.79–0.81)	0.43 (0.42–0.46)	0.91 (0.88–0.93)	0.59 (0.59–0.60)	0.75 (0.74–0.76)	0.43 (0.42–0.43)
Light-GBM	0.87 (0.86–0.89)	0.20 (0.18–0.24)	0.55 (0.51–0.58)	0.91 (0.91–0.92)	0.73 (0.71–0.75)	0.60 (0.59–0.61)
RF	0.79 (0.77–0.80)	0.34 (0.32–0.36)	0.75 (0.72–0.78)	0.69 (0.69–0.7)	0.72 (0.71–0.74)	0.50 (0.50–0.50)
MLP	0.86 (0.85–0.87)	0.19 (0.17–0.22)	0.36 (0.33–0.39)	0.96 (0.96–0.97)	0.66 (0.66–0.68)	0.63 (0.61–0.64)
KNN	0.75 (0.73–0.77)	0.18 (0.16–0.20)	0.14 (0.11–0.16)	0.98 (0.98–0.99)	0.56 (0.55–0.57)	0.57 (0.56–0.58)
DC	0.51 (0.49–0.53)	0.28 (0.26–0.30)	0.52 (0.48–0.55)	0.50 (0.50–0.51)	0.51 (0.49–0.53)	0.36 (0.36–0.36)

*AUC* Area under the curve, *AUCPR* Area under the precision recall curve, *LR* Logistic regression, *SVM* Support vector machine, *NB* Naive bayes, *LightGBM* Light gradient boosting machine, *RF* Random forest, *MLP* Multilayer perceptron, *KNN* K-nearest neighbors, *DC* Dummy classifier

The DCA results demonstrate that across the full range of threshold probabilities, all three models exhibited a higher net benefit compared to considering all patients as cases. Moreover, the net benefits of all three models showed similar patterns (Figure S4).

### Important feature selection

A total of 250 rounds of permutation importance was performed to retrieve the most influential variables from SVM, LR and Xgboost based on their contribution to the balanced accuracy (Figures S1, S2 and S3). Age was found to be in the top two most predictive variables for in-hospital mortality in SVM (mean balanced accuracy ± standard deviation 0.047 ± 0.006, rank 2), LR (0.054 ± 0.006, rank 1) and Xgboost (0.055 ± 0.006, rank 1) models. For SVM and LR a total of 10, and for Xgboost a total of nine variables were strongly associated with in-hospital mortality (Table 3).

**Table 3:**
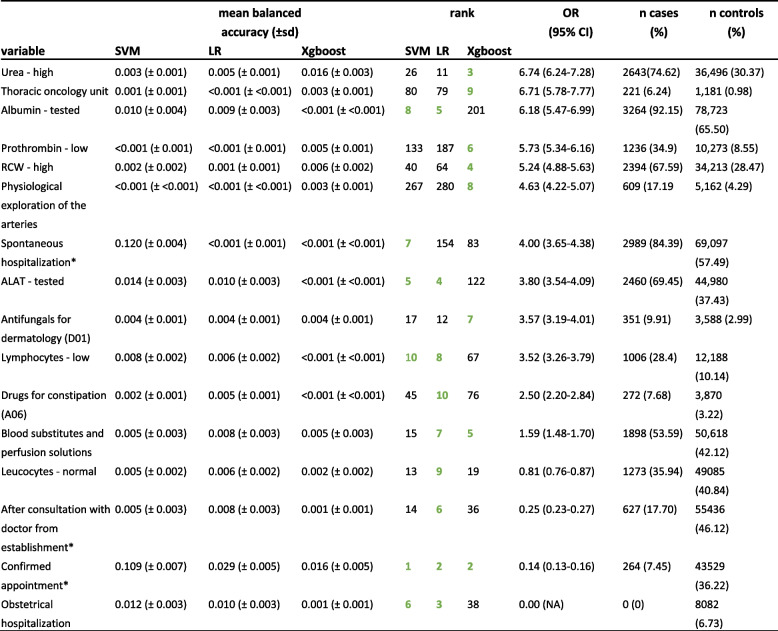
Most influential categorical variables for support vector machine (SVM), logistic regression (LR) and Xgboost from 250 rounds of permutation importance, sorted by odds ratio

Selected variables in each model are highlighted in bold green. All variables show a *p*-value of < 0.0001 (chi-square test). Alanine-aminotransferase (ALAT), aspartate-aminotransferase (ASAT), red cell distribution width (RCW). *Modes of admission: “After consultation with doctor from the establishment* is when a patient is seen in a scheduled consultation and kept in hospital immediately afterwards, without going through the emergency room, therefore without immediate vital risk; whereas “Confirmed prescheduled appointment” is hospitalization programmed in advance, e.g., for non-urgent surgery, again without immediate vital risk

**Fig. 4:**
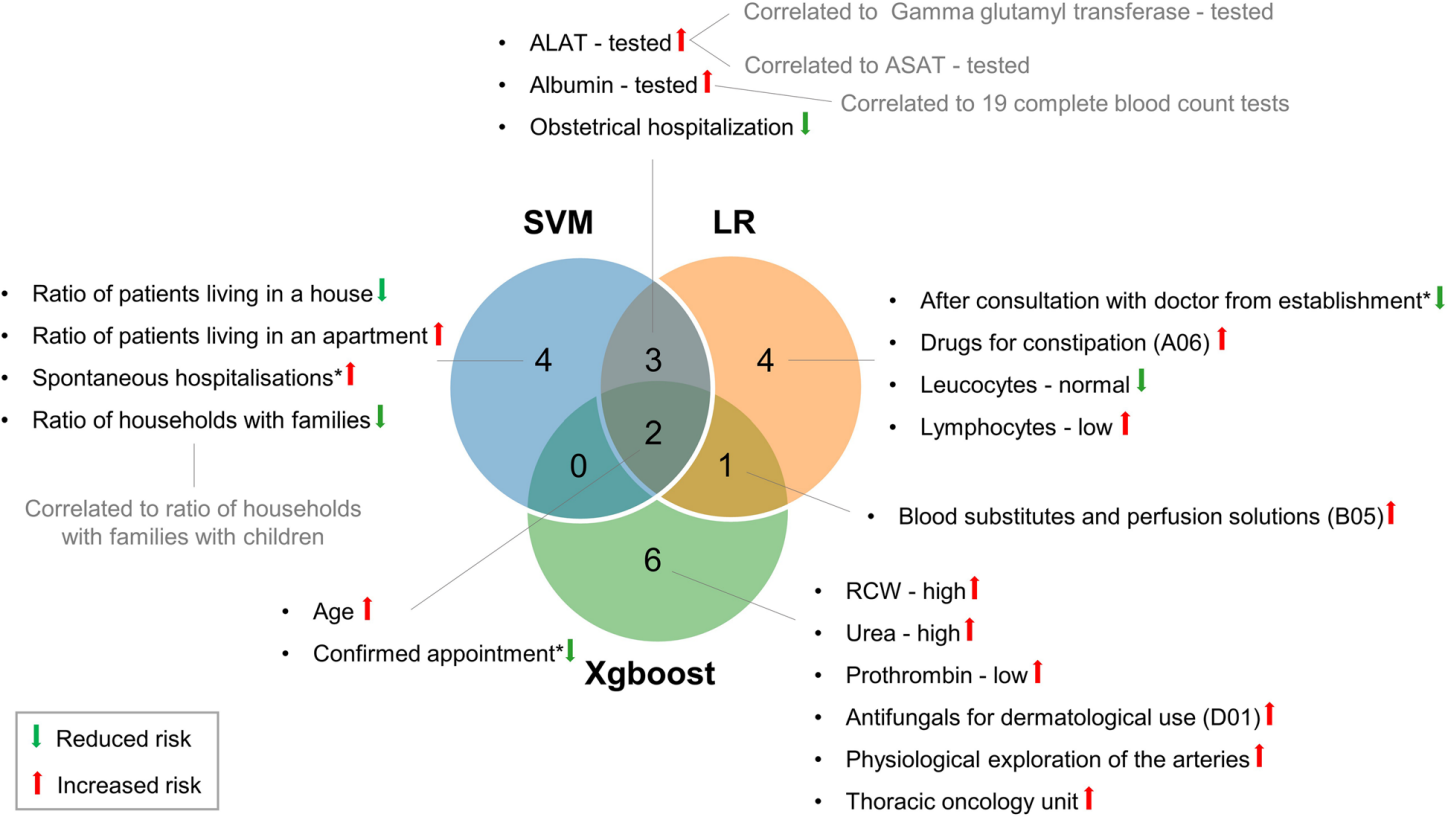
Overlapping variables between support vector machine (SVM), logistic regression (LR) and Xgboost. Alanine-aminotransferase (ALAT), aspartate-aminotransferase (ASAT), red cell distribution width (RCW). *Modes of admission: “After consultation with doctor from the establishment”* is when a patient is seen in a scheduled consultation and kept in hospital immediately afterwards, without going through the emergency room, therefore without immediate vital risk; whereas “Confirmed prescheduled appointment” is hospitalization programmed in advance, e.g., for non-urgent surgery, again without immediate vital risk

Overall, age and being admitted to the hospital with a confirmed appointment were identified in all three models as crucial (Fig. 4). Although, the rank of variable importance varied depending on the machine learning model, some variables ranked consistently high in all three models.

We noted that testing alanine-aminotransferase (ALAT) was highly correlated with testing other liver enzymes (Gamma glutamyl transferase and aspartate-aminotransferase (ASAT); Pearson correlation coefficient = 1.0) (Table S4 (a) and (c)). Testing for plasma albumin was correlated with testing for 19 variables from the complete blood count (Table S4 (a) (c)). In addition, the SVM model considered three social determinants of health as important. These were the “patients living in an apartment” (mean balanced accuracy 0.046 (± 0.006), rank 3, higher in cases), “patients living in a house” (0.038 (± 0.005), rank 4, lower in cases) and “households with families” (0.010 (± 0.003), rank 9, lower in cases). These also showed significant differences between cases and controls (*p*-value < 0.05). The socio-economic variable “households with families” was highly correlated with the socio-economic variable “households with families with children” (Pearson correlation coefficient = 0.9) (Table S4 (b)).

## Discussion

The main aim of this study was to generate a predictive model for in-hospital mortality based on data from the PREDIMED clinical data warehouse [23] along with publicly available socio-economic variables. In contrast to other studies, we focused our analyses not on a specific subgroup of patients but on the general adult population admitted to our University Hospital, which makes our model applicable to a wide range of patients admitted to large teaching hospitals in France and possibly elsewhere in Europe. In addition, this study utilized a wide range of types of variables, which have not been included in previous studies.

We succeeded in generating highly discriminating prediction models for in-hospital mortality. The models identified multiple plausible risk factors and risk protectors at the time of admission. Older age is a well-known risk factor for mortality; already identified in two studies using machine learning, one in emergency departments to predict early and short-term mortality [16] and the other in the University of Tokyo hospital to predict in-hospital mortality within 14 days [10]. In contrast, being admitted to the hospital with a confirmed appointment i.e., being admitted without a newly emergent issue, was a low-risk factor for in-hospital mortality. Several clinical scores, such as SAPS II, have used the patient’s location before ICU admission to predict mortality [17]. In this study, among the variables we used for building the machine learning models several laboratory parameters were found to be important in predicting in-hospital mortality. Risk factors were high ALAT, high albumin, high urea, low lymphocytes, and low prothrombin. A low-risk factor was a normal leucocyte count. These anormal biological laboratory analysis results are markers of serious pathologies. High ALAT reflects liver pathology, high albumin and high urea a state of dehydration or hemo-concentration and renal failure. Low lymphocytes expose the risk of infection and low prothrombin reflects either liver failure or the presence of anticoagulant for cardiovascular problems. In line with this, it has been previously reported that these parameters were markers for predicting the prognosis of hospitalized patients, and this indicates that our internal algorithms of the prediction models are consistent with current evidence [10, 11, 17]. Of note, high red cell distribution width was recently found to be a risk factor for mortality after Covid-19 [43]. Other consistent risk factors were the administration of blood substitutes and perfusion solutions, physiological exploration of the arteries, and hospitalization in a thoracic oncology unit. A consistent low-risk factor was hospitalization in an obstetrics unit.

Use of drugs for constipation, antifungals for dermatological use, whether the patient lives in a house or an apartment or in a household with a family, are unusual predictive factors which require additional investigation.

In a next step the models need to be validated using more recent data from our hospital and finally in everyday clinical practice. Up to now, no predictive model for all adult patient in-hospital mortality has been validated in clinical practice.

The hospital (CHUGA) is a large tertiary teaching hospital with a sizeable trauma unit, and is particularly active in highly specialized procedures and in clinical care. The model is unlikely to be applicable to hospitals with a different profile and different coding scheme. Overall, nine different machine learning algorithms were compared, showing that regression algorithms (such as LR) and instance-based algorithms (such as SVM) were superior to Bayesian algorithms (NB), RF, deep learning algorithms (MLP) and non-parametric algorithms (KNN). SVM, LR and Xgboost demonstrated high predictive ability with good balanced accuracy (Table 2) when the full set of variables was used. Indeed, the alignment of model performances derived from LR, SVM, and Xgboost, each utilizing different sets of variables, points to the comprehensive tuning of hyperparameters. Interestingly, while the three methods exhibit comparable performance, it is noteworthy that LR and SVM, both representing linear models, show a closer alignment in terms of feature overlap. This alignment hints at the nature of mortality prediction as a linear classification task, where these linear models prove to be as effective as even more intricate models like Xgboost. Moreover, all these models outperformed the dummy classifier which was used as a benchmark model. Regarding the achieved AUC (Table 2), our results are similar [19] or slightly lower than in other studies (AUC ~ 0.95) [10, 11, 16]. Notably, KNN was inferior in predicting in-hospital mortality but showed superior specificity compared to LR, SVM and Xgboost. Since our Xgboost model requires the smallest number of variables (45 vs. 75 in LR and SVM) to achieve similar model performances compared to using all variables, this model is thus most suitable for application in clinical practice. As our models would allow physicians to increase their focus on patients at risk, which would also increase the quality of care in general, the relatively high number of false positives is not necessarily a disadvantage. Moreover, our top three models demonstrate high sensitivities, correctly identifying positive cases and reducing false negatives compared to other models in the study. Models with the highest specificity, namely Light-GBM, MLP, and KNN, achieved the highest F1-scores. Nevertheless, this gain in specificity is accompanied by a decrease in sensitivity. Overall, the low number in false positives could help to optimize healthcare resource allocation and minimize unnecessary interventions and associated costs. Overall, our model’s ability to tackle alarm fatigue underscores their high value as potential tools in medical decision-making and patient care.

Our DCA demonstrates that across the entire range of threshold probabilities, all three models (SVM, LR, and Xgboost) consistently exhibit higher net benefits compared to the strategy of considering all patients as cases. This finding highlights the clinical value of these predictive models, as they consistently outperform the simple approach of treating all patients as positive cases, even when accounting for different decision thresholds. Furthermore, the DCA results indicate that the three models have very similar net benefits throughout the entire threshold range. This similarity underscores the robustness of their performance and suggests that they are reliable in a wide range of decision-making scenarios.

Model performance might be improved in the future if more data becomes available, and the coding behavior is further standardized. To date, we could only consider diagnoses from the patient’s previous hospitalization (if any) because of inadequate time stamps. We could not use any unstructured information from the electronic health records. However, including these might further benefit the models’ performances. The models’ performances might be even more improved by additional algorithm hyperparameter tuning and optimization.

Our study has several limitations. First, we chose to censor in-hospital mortality at 30 days. The 30-day delay is the usual delay in public health studies. We had no information on deaths after discharge from hospital, so we cannot rule out that some patients died within 30-days in another establishment or at home. Moreover, we cannot distinguish between patients who enter the hospital with an end-stage disease and those who die as a consequence of an intervention during their hospitalization, or a complication acquired during their hospitalization. Second, we only analyzed the data recorded in the patients’ files. Third, there was no updating of the patients’ EMR data during hospitalization, whereas medical staff usually routinely record important comorbidities or changes in medical condition. Forth, laboratory analysis values were not available for all patients. Nevertheless, biological parameters such as albumin were usually requested in cases in which abnormalities were suspected. Fifth, we lack information from other establishments about previous hospitalizations. Sixth, the decision to generate a single prediction at the time of inpatient admission and only use data available up to two days after admission might be subject to bias when evaluating the mortality at medium term (up to 30 days after admission). In some rare cases, a patient could be in relatively good health at hospital admission and their health subsequently deteriorate very quickly until death. Finally, additional research is required to assess the generalizability of our results to other settings.

Nevertheless, our study has several strengths. What distinguishes our research is its ability to establish a prospective risk assessment for individual patients within the first day of admission, encompassing a broad spectrum of variables. This permits appropriate care pathways to be implemented from the first few days of hospitalization onwards. While many investigations tend to focus on specific patient cohorts, our study addresses the entire hospitalized patient population. This differentiation lies in our holistic approach, which incorporates diverse variables, including clinical, administrative, and social determinants of health. This multifaceted perspective enables us to provide a personalized and comprehensive risk assessment for each patient, marking a significant contribution to the field. Additionally, our methodological approach strikes a balance between model complexity, data size, and practical applicability in medical practice, aligning with best practices in data science and EHR data modelling. Critical to our approach were meticulous data exploration and variable extraction, ensuring the relevance of information while guarding against overfitting in machine learning models. These steps underpin the robustness and clinical utility of our approach. Moving forward, we plan to validate our models with an expanded dataset from additional hospitals and leverage them in the development of a warning system. This system will alert physicians to patients at risk, ultimately enhancing healthcare resource allocation and management.

## Conclusion

Our highly discriminating prediction models identified multiple risk factors for in-hospital mortality in the data available within the first full day of admission. Our extraction of the most impactful variables for LR and SVM, will enable physicians to understand which information has been used by the algorithms. This will improve the acceptance of predictive models in everyday practice.

The routine use of predictive models that alert healthcare professionals and administrators to a patient´s heightened risk of dying within the next 30 days has the potential to improve efficient resource management and augment the monitoring of patients most at risk.

### Supplementary Information

**Additional file 1.**

## Data Availability

The de-identified raw data supporting the reported results are not publicly available as they contain the complete medical files of all patients included in the database, so for ethical reasons they cannot be readily exported to others. Please see https://www.chu-grenoble.fr/patients-et-accompagnants/la-recherche-au-chuga/entrepot-de-donnees-de-sante-eds. However, reasonable justified requests for specific data should be made to the corresponding author and to protection-donnees@chu-grenoble.fr. Restrictions apply to the availability of these data: “Any project that requires access to a subset of CDW data follows a strict evaluation procedure by a set of local committees. This involves ensuring the admissibility of the project by a methodological, regulatory, technical and financial evaluation carried out by the Project Steering Committee as well as by a scientific and then ethical and deontological evaluation carried out by dedicated committees. Finally, any provision of data within the framework of a project (which complies with the medico-legal procedures in force and validated by the governance) is subject to a final verification by an independent appointed committee.”

## Abbreviations

ATC: Anatomical Therapeutic Chemical code
AUC: Area under the curve
AUCPR: Area under the precision recall curve
CDW: Clinical Data Warehouse
CHUGA: Grenoble Alpes University Hospital
CI: Confidence interval
CMA: Classification of Medical Acts
DC: Dummy classifier
DCA: Decision curve analysis
EHR: Electronic health records
FDR: False discovery rate
FN: False negative
FP: False positive
INSEE: French national institute for statistical and economic studies
KNN: K-nearest neighbors
LightGBM: Light gradient boosting machine
LR: Logistic Regression
MLP: Multilayer Perceptrons
NB: Naive Bayes
RF: Random Forest
ROC: Receiver operating characteristic
SDOH: Social determinants of health
SVM: Support Vector Machines
TN: True negative
TP: True positive
