# Supplementary material for: Early prediction of in-hospital mortality utilizing multivariate predictive modelling of electronic medical records and socio-determinants of health of the first day of hospitalization
Source: BMC Med Inform Decis Mak. 2023 Nov 13;23:259. doi: 10.1186/s12911-023-02356-4 (PMC10644472; doi:10.1186/s12911-023-02356-4)
Supplement: Supplementary file 1 — Additional file 1. [file 12911_2023_2356_MOESM1_ESM.pdf]

Early prediction of in-hospital mortality utilizing multivariate predictive modelling of electronic medical records and socio-determinants of health of the first day of hospitalization

Daniel Stoessel, Rui Fa, Svetlana Artemova, Ursula von Schenck, Hadiseh Nowparast Rostami, Pierre-Ephren Madiot, Caroline Landelle, Fréderic Olive, Alison Foote, Alexandre Moreau-Gaudry, Jean-Luc Bosson

Supplementary material

Contents

Supplementary Tables

S1: Distribution of various Social Determinants of Health (SDOH) between cases (hospital death) and controls (no in-hospital death) in days 3 to 30
S2 (a): Parameter levels used for model algorithm hyperparameter optimization
S2 (b): Optimized algorithm hyperparameters
S3 (a): Levels of preselected variables
S3 (b): Number of medications in the observation time frame
S4 (a): Correlation analysis categorical variables (Pearson correlation coefficient >0.9)
S4 (b): Correlation analysis continuous variables (Pearson correlation coefficient >0.9)
S4 (c): Categories for correlated lab tests (Pearson correlation coefficient >0.9)
S5 (a): All primary diagnoses from cases and controls during their hospitalization with an FDR of <0.01, ordered by odds ratio
S5 (b): All secondary diagnoses from cases and controls during their hospitalization with an FDR of <0.01, ordered by odds ratio

Supplementary Figures

Figure S1: Top 75 most influential variables sorted by their contributing mean balanced accuracy for the support vector machine algorithm. Error bars indicate standard deviation based on 250 rounds of permutation importance. Dashed line shows the threshold used as a cut-off for the most important variables, based on the area where the slope of the curve flattens.
Figure S2: Top 75 most influential variables sorted by their contributing mean balanced accuracy for the logistic regression algorithm. Error bars indicate standard deviation based on 250 rounds of permutation importance. Dashed line shows the threshold used as a cut-off for the most important variables, based on the area where the slope of the curve flattens.
Figure S3: Top 45 most influential variables sorted by their contributing mean balanced accuracy for the xgboost algorithm. Error bars indicate standard deviation based on 250 rounds of permutation importance. Dashed line shows the threshold used as a cut-off for the most important variables, based on the area where the slope of the curve flattens.
Figure S4: Decision curve analysis for the top three performing models using the full set of variables.

Table S1. Distribution of various Social Determinants of Health (SDOH) between cases (hospital death) and controls (no in-hospital death) in days 3 to 30

Category	Variable code	Variable definition	mean case	mean control	std case	std control	max case	max control	25%* case	25%* control	50%* case	50%* control	75%* case	75%* control
Age Group	P18_POP0014	number of people aged 0 to 14	0.18	0.18	0.04	0.04	0.31	0.44	0.16	0.16	0.18	0.18	0.20	0.20
	P18_POP1529	number of people aged 15 to 29	0.19	0.19	0.08	0.07	0.59	0.98	0.14	0.14	0.17	0.16	0.20	0.20
	P18_POP3044	number of people aged 30 to 44	0.18	0.19	0.03	0.03	0.30	0.39	0.17	0.17	0.18	0.18	0.20	0.20
	P18_POP4559	number of people aged 45 to 59	0.20	0.20	0.03	0.04	0.38	0.43	0.18	0.18	0.20	0.20	0.22	0.22
	P18_POP6074	number of people aged 60 to 74	0.16	0.16	0.04	0.04	0.41	0.47	0.13	0.13	0.15	0.15	0.18	0.18
	P18_POP75P	number of people aged 75 or over	0.10	0.09	0.03	0.04	0.25	0.62	0.07	0.07	0.09	0.09	0.11	0.11
Population Structure	P18_POP_IMM	immigrent population	0.11	0.11	0.08	0.08	0.49	0.63	0.06	0.05	0.09	0.09	0.14	0.14
	C18_POP15P_CS1	pop 15 years or more, farmer operators	0.00	0.00	0.01	0.01	0.17	0.40	0.00	0.00	0.00	0.00	0.00	0.00
	C18_POP15P_CS2	pop 15 years or more, craftsmen etc.	0.04	0.04	0.02	0.02	0.31	0.32	0.02	0.02	0.03	0.03	0.04	0.04
	C18_POP15P_CS3	pop 15 years or over executives. prof. intel. sup.	0.12	0.12	0.07	0.07	0.37	0.71	0.07	0.06	0.11	0.10	0.17	0.15
	C18_POP15P_CS4	pop 15 years or over intermediate occupation	0.15	0.15	0.04	0.04	0.30	0.42	0.13	0.13	0.15	0.15	0.17	0.17
	C18_POP15P_CS5	pop 15 years or over, salaried employees	0.15	0.15	0.04	0.04	0.43	0.48	0.13	0.13	0.15	0.15	0.18	0.18
	C18_POP15P_CS6	pop 15, years or over, workers	0.10	0.11	0.05	0.05	0.30	0.62	0.07	0.07	0.10	0.10	0.13	0.14
	C18_POP15P_CS7	pop 15 years or over, retired	0.26	0.26	0.07	0.07	0.76	0.83	0.21	0.21	0.26	0.25	0.29	0.30
	C18_POP15P_CS8	pop 15 years or over, others	0.17	0.17	0.06	0.07	0.65	0.90	0.13	0.13	0.16	0.16	0.19	0.19
Family	C18_MENPSEUL	number of one-person households	0.37	0.37	0.11	0.11	0.82	1.00	0.29	0.29	0.36	0.35	0.44	0.44
	C18_MENSFAM	number of other households without family	0.02	0.02	0.02	0.02	0.15	0.25	0.01	0.01	0.02	0.02	0.03	0.03
	C18_MENFAM	number of households with family(ies)	0.60	0.61	0.12	0.12	0.87	1.00	0.53	0.54	0.62	0.62	0.69	0.70
	C18_MENCOUPSENF	households whose main family is a couple without children	0.26	0.26	0.07	0.07	0.85	0.85	0.21	0.22	0.25	0.26	0.30	0.31
	C18_MENCOUPAENF	households where main family is a couple with children	0.25	0.26	0.08	0.08	0.53	0.67	0.20	0.21	0.25	0.26	0.31	0.31
	C18_MENFAMMONO	households where main family is a single-parent family	0.09	0.09	0.03	0.03	0.25	0.33	0.07	0.07	0.09	0.09	0.11	0.11
	P18_POP1524_PSEUL	number of people aged 15 to 24, living alone	0.02	0.02	0.03	0.03	0.36	0.62	0.00	0.00	0.01	0.01	0.02	0.01
	P18_POP2554_PSEUL	number of people aged 25 to 54, living alone	0.06	0.06	0.03	0.03	0.20	0.43	0.04	0.04	0.05	0.05	0.07	0.07

Category	Variable code	Variable definition	mean case	mean control	std case	std control	max case	max control	25%* case	25%* control	50%* case	50%* control	75%* case	75%* control
Family	P18_POP5579_PSEUL	number of people aged 55 to 79, living alone	0.07	0.07	0.02	0.02	0.19	0.39	0.05	0.05	0.07	0.06	0.08	0.08
	P18_POP80P_PSEUL	number of people aged over 80, living alone	0.03	0.03	0.01	0.01	0.10	0.62	0.02	0.02	0.02	0.02	0.03	0.03
Housing	P18_MAISON	number of houses	0.40	0.42	0.32	0.32	1.00	1.00	0.09	0.10	0.32	0.38	0.69	0.73
	P18_APPART	number living in appartments	0.60	0.58	0.32	0.32	1.00	1.00	0.31	0.27	0.68	0.61	0.91	0.90
	P18_RP_1P	main residence: 1 room	0.05	0.04	0.06	0.06	0.61	0.93	0.01	0.01	0.02	0.02	0.05	0.05
	P18_RP_2P	main residence: 2 rooms	0.10	0.10	0.06	0.06	0.34	0.55	0.06	0.05	0.10	0.09	0.13	0.13
	P18_RP_3P	main residence: 3 rooms	0.20	0.19	0.08	0.08	0.45	0.58	0.14	0.13	0.20	0.20	0.25	0.25
	P18_RP_4P	main residence 4 rooms	0.24	0.24	0.08	0.08	0.51	0.59	0.19	0.19	0.25	0.25	0.28	0.28
	P18_RP_5PP	main residence 5 rooms or more	0.28	0.28	0.16	0.16	0.73	0.89	0.15	0.15	0.24	0.25	0.39	0.41
	P18_RP_ACH19	main residence built before 1919	0.07	0.08	0.07	0.08	0.47	0.83	0.01	0.01	0.05	0.05	0.11	0.11
	P18_RP_ACH45	main residence 1built 919 to 1945	0.05	0.05	0.04	0.04	0.29	0.53	0.02	0.02	0.04	0.04	0.06	0.06
	P18_RP_ACH70	main residence built 1946 to 1970	0.23	0.21	0.16	0.15	0.83	0.85	0.11	0.10	0.19	0.18	0.32	0.26
	P18_RP_ACH90	main residence built 1971 to 1990	0.26	0.26	0.12	0.12	0.90	1.00	0.19	0.18	0.25	0.25	0.31	0.31
	P18_RP_ACH05	main residence built 1991 to 2005	0.13	0.14	0.08	0.08	0.70	0.73	0.07	0.07	0.12	0.13	0.18	0.18
	P18_RP_ACH15	main residence built 2006 to 2015	0.10	0.10	0.08	0.08	0.47	0.89	0.04	0.05	0.08	0.09	0.13	0.14
	P18_RP_PROP	main residence owner occupied	0.51	0.51	0.18	0.18	0.88	0.93	0.38	0.37	0.54	0.53	0.65	0.65
	P18_RP_LOC	main residence rented	0.34	0.34	0.17	0.17	0.95	1.03	0.21	0.20	0.34	0.33	0.45	0.45
	P18_RP_LOCHLMV	main residence social housing rented empty	0.13	0.13	0.14	0.14	0.93	0.93	0.04	0.04	0.08	0.08	0.17	0.16
	P18_RP_GRAT	main residence occupied for free	0.02	0.02	0.02	0.02	0.25	1.00	0.01	0.01	0.02	0.02	0.02	0.02
	P18_RP_SDB	main residence has bathroom	0.85	0.84	0.12	0.14	1.04	1.38	0.82	0.82	0.89	0.88	0.91	0.91
	P18_RP_VOIT1	households with one car	0.42	0.41	0.09	0.10	0.67	1.00	0.35	0.34	0.44	0.43	0.48	0.48
	P18_RP_VOIT2P	households with 2 cars or more	0.31	0.31	0.16	0.16	0.65	0.74	0.18	0.18	0.29	0.30	0.44	0.45
Activity	P18_CHOM1564	unemployed 15-64 years	0.09	0.09	0.04	0.04	0.30	0.37	0.07	0.07	0.09	0.09	0.11	0.11
	P18_CHOM1524	unemployed 15-24 years	0.02	0.02	0.01	0.01	0.08	0.12	0.01	0.01	0.02	0.02	0.02	0.03
	P18_CHOM2554	unemployed 25-54 years	0.06	0.06	0.03	0.03	0.21	0.30	0.04	0.04	0.06	0.06	0.07	0.07
	P18_CHOM5564	unemployed 55-64 years	0.01	0.01	0.01	0.01	0.06	0.11	0.01	0.01	0.01	0.01	0.01	0.01
	P18_HINACT1564	inactive men 15-64 years	0.12	0.12	0.03	0.03	0.57	1.00	0.11	0.11	0.12	0.12	0.14	0.14
	P18_FINACT1564	inactive women 15-64 years	0.15	0.15	0.03	0.04	0.33	0.52	0.13	0.13	0.15	0.15	0.17	0.17
	P18_RETR1564	retired/early retirement aged 15-64	0.06	0.07	0.03	0.03	0.23	1.00	0.04	0.05	0.06	0.06	0.08	0.08

Minimum case and control values are always 0.00; *25% and 75% percentiles and 50% percentile (median)

S2 (a): Parameter levels used for model algorithm hyperparameter optimization

Algorithm	Hyperparameter	Range	Optimization rounds
Xgboost	n estimators	"10-100"	300
	eta	"0.025-0.5"	
	max depth	"1-5"	
	min child weight	"1-10"	
	subsample	"0.1-0.5"	
	gamma	"0.1-0.5"	
	colsample bytree	"0.1-0.5"	
	reg lamda	"0-1"	
	colsample bylevel	"0.1-0.5"	
LightGBM	n estimators	100	300
	max depth	"5-15"	
	subsample	0.8, 1	
	colsample bytree	"0.3-0.7"	
	learning rate	0.05-0.30	
	min child weight	"1-7"	
RF	criterion	entropy	250
	n estimators	10, 20, 30, 40, 50, 60, 70	
	max features	log2	
	min samples leaf	1, 2, 3, 4, 5, 6, 7	
	ccp alpha	0.1, 0.2, 0.3, 0.4, 0.5, 0.6, 0.7	
	bootstrap	True	
KNN	n_neighboors	10-100	20
SVM	kernel	linear	300
	C	0.001-1	
LR	solver	liblinear, saga, newton-cg, lbfgs, sag	300
	penalty	l1, l2	
	max_iter	0.00001-0.0001	
	tol	0.00001-0.0001	
	C	0.001-1	
NB	/	/	/
DC	strategy	stratified	/
MLP	number hidden layers	3, 4, 5	1000
	number neurons	100, 250, 500, 750, 1000	
	activation	elu, selu, sigmoid, linear, tanh, relu	
	optimizer	adam, rmsprop, adagrad, nesterov momentum	
	batch size	100, 250, 500	
	learning rate	$1x10^{-6}$ - $1x10^{-2}$	
	dropout	0.2-0.6	

LR: Logistic Regression; SVM: Support Vector Machine; NB: Naive Bayes; RF: Random Forest; LightGBM: light gradient boosting machine deep learning; MLP: algorithms such as Multilayer Perceptrons; KNN: k-nearest neighbors; DC: dummy classifier.

S2 (b): Optimized algorithm hyperparameters

Algorithm	Hyperparameter	Optimized value	Optimization rounds
Xgboost	n estimators	96	300
	eta	0.275	
	max depth	4	
	min child weight	1	
	subsample	0.5	
	gamma	0.300	
	colsample bytree	0.5	
	reg lamda	0.750	
	colsample bylevel	0.45	
LightGBM	n estimators	100	300
	max depth	12	
	subsample	0.964	
	colsample bytree	0.6	
	learning rate	0.30	
	early stopping rounds	10	
	min child weight	6	
RF	criterion	entropy	250
	n estimators	40	
	max features	log2	
	min samples leaf	2	
	ccp alpha	0.1	
	bootstrap	True	
KNN	n_neighboors	10	20
SVM	kernel	linear	300
	C	0.681	
LR	solver	lbfgs	300
	penalty	l2	
	max_iter	432.410	
	tol	5.8487x10-5	
	C	0.060	
NB	/	/	/
MLP	number hidden layers	3	1000
	number neurons	750	
	activation	relu	
	optimizer	adam	
	batch size	100	
	learning rate	0.0036	
	dropout	0.200	

LR: Logistic Regression; SVM: Support Vector Machine; NB: Naive Bayes; RF: Random Forest; LightGBM: light gradient boosting machine deep learning; MLP: algorithms such as Multilayer Perceptrons; KNN: k-nearest neighbors.

Table S3 (a). Number and percentage of selected variables for cases (admissions with in-hospital death) and controls (no in-hospital death between day 3 and day 30).

For some categories not all possible variables are presented, or the information was not available.

Variable category	Variable code and/or description	Cases N = 3542	Controls N= 120.187	% cases	% controls
Entry Mode* (mode of admission)	after consultation with doctor from establishment	627	55436	17.7	46.12
	other mode of entry	332	6176	9.37	5.14
	from home	2212	51816	62.45	43.11
	transfer from another establishment	271	4973	7.65	4.14
Entry Type*	confirmed appointment	264	43536	7.45	36.22
	spontaneous	2989	69097	84.39	57.49
	urgent	289	7554	8.16	6.29
Hospital Department	2170 (ICU)	225	2142	6.35	1.78
	3610 (thoracic oncology unit)	221	1181	6.24	0.98
	3790 (ICU)	238	1254	6.72	1.04
	3836 (emergency unit)	1981	37609	55.93	31.29
	3838 (emergency unit)	620	8668	17.5	7.21
	8280 (ICU)	211	1961	5.96	1.63
	9290 (obstetrical hospitalization)	0	8083	0	6.73
Diagnosis from previous hospital stay	i10 (essential (primary) hypertension)	413	10885	11.66	9.06
	i48 (atrial fibrillation and flutter)	218	3954	6.15	3.29
	z92 (personal history of medical treatment)	178	4861	5.03	4.04
Laboratory tests (tested)	plasma ALT (GPT)_ui/l	2460	44980	69.45	37.43
	albumin_g/l	3299	79793	93.14	66.39
	basophils_g/l	3266	78831	92.21	65.59
	bicarbonates - total CO2_mmol/l	3302	75336	93.22	62.68
	bicarbonates_mmol/l	3302	75336	93.22	62.68
	conjugated bilirubin_µmol/l	3264	72742	92.15	60.52
	total bilirubin_µmol/l	3264	72742	92.15	60.52
	calcium_mmol/l	3302	75336	93.22	62.68
	mean corpuscular hemoglobin concentration (MCHC)_g/l	3299	79793	93.14	66.39
	chlorides_mmol/l	3098	69665	87.46	57.96
	blood O2 content _vol%	1397	13557	39.44	11.28
	plasmatic creatine-kinase (ck)_ui/l	1413	25416	39.89	21.15
	creatinine_µmol/l	3264	72742	92.15	60.52
	c-reactive protein (crp)_mg/l	2740	55497	77.36	46.18
	eosinophils_g/l	3266	78831	92.21	65.59
	erythrocytes_t/l	3266	78830	92.21	65.59
	base excess_mmol/l	3302	75336	93.22	62.68
	factor II_%	3285	80502	92.74	66.98
	factor V_%	3285	80502	92.74	66.98
	factor X_%	3285	80502	92.74	66.98
	plasmatic GGT (gamma glutamyl transferase)_ui/l	2460	44980	69.45	37.43
	glycemia_mmol/l	3302	75336	93.22	62.68
	hemoglobin (measure)_g/l	3299	79793	93.14	66.39
	hematocrit_l/l	3266	78829	92.21	65.59
	Red blood cell distribution width (RDW)_%	3285	80502	92.74	66.98

Variable category	Variable code and/or description	Cases N = 3542	Controls N= 120.187	% cases	% controls
Laboratory tests (tested)	plasma lactate dehydrogenase (ldh)_ul/l	1611	26821	45.48	22.32
	lactate_mmol/l	3302	75336	93.22	62.68
	leucocytes_g/l	3266	78831	92.21	65.59
	plasmatic lipase_ui/l	1413	25416	39.89	21.15
	lymphocytes #_g/l	3266	78831	92.21	65.59
	mono #_g/l	3266	78831	92.21	65.59
	neutro #_g/l	3266	78831	92.21	65.59
	osmolarity (c)_mosmol/l	1508	30338	42.57	25.24
	pCO2_kpa	1412	13728	39.86	11.42
	plasma alkaline phosphatase_ul/l	1611	26821	45.48	22.32
	phosphorus_mmol/l	3302	75336	93.22	62.68
	pO2_kpa	1412	13728	39.86	11.42
	plasmatic potassium (k)_mmol/l	3302	75336	93.22	62.68
	Pre-albumin_mg/l	2740	55497	77.36	46.18
	plasmatic proteins_g/l	2961	65509	83.6	54.51
	reticulocytes_g/l	3266	78831	92.21	65.59
	sodium (Na)_mmol/l	3302	75336	93.22	62.68
	prothrombin rate_%	3285	80502	92.74	66.98
	mean cell hemoglobin (MCH)_pg	3265	78767	92.18	65.54
	prothrombin time_s	1439	31719	40.63	26.39
	plasmatic sgot (ast)_ui/l	2460	44980	69.45	37.43
	plasmatic uric acid_mmol/l	3080	68162	86.96	56.71
	thrombocytes_g/l	3266	78831	92.21	65.59
	cardiac troponin i_µg/l	1863	27225	52.6	22.65
	urea_mmol/l	3302	75336	93.22	62.68
	mean corpuscular volume_fl	3266	78829	92.21	65.59
Procedure Code	acqk (cranial and brain scan)	640	8989	18.07	7.48
	deqp (electrocardiogram)	1962	32271	55.39	26.85
	dzqm (functional heart imaging)	255	2146	7.2	1.79
	ecqh (arteriography)	254	3655	7.17	3.04
	enlf (application of a blood pressure device)	435	2098	12.28	1.75
	eplf (central venous catheter insertion)	481	2284	13.58	1.9
	eqlf (intravenous injection of vasopressor drugs)	1071	11029	30.24	9.18
	eqqp (physiological exploration of the arteries)	609	5162	17.19	4.29
	geld (quite different according to the last three numbers: 002-004 tracheal intubation, 001 and 005 continuous aerosol)	191	1570	5.39	1.31
	glhf (arterial blood gasometry)	693	6506	19.57	5.41
	glld (external ventilation methods)	896	10134	25.3	8.43
	jdld (001 bladder catheterization ; intravesical therapy by transurethral route)	279	3599	7.88	2.99
	yyyy (technical gesture complementary to a diagnostic act)	1737	40545	49.04	33.73
	zbqk (imaging of the thorax without injection of contrast medium)	1304	19343	36.82	16.09
	zcqh (abdomino-pelvic imaging without injection of contrast medium)	326	6405	9.2	5.33

Variable category	Variable code and/or description	Cases N = 3542	Controls N= 120.187	% cases	% controls
Procedure Code	zcqk (abdomino-pelvic imaging without injection of contrast medium)	201	2534	5.67	2.11
	zzqk (multi-incidence imaging)	857	12007	24.2	9.99
	zzqm (ultrasound scan)	230	2796	6.49	2.33
	zzqp (quite different according to the last three numbers: 001 and 003 medical monitoring of patient during transport, 004 three-dimensional restitution of images acquired by ct scan)	1300	20989	36.7	17.46
	zzqx (immunhistology with 3 to 5 antibodies)	276	12468	7.79	10.37
Medication	a02 (drugs for acidocis and related disorders)	1363	40312	38.48	33.54
	a03 (drugs for functional gastrointestinal disorders)	644	18624	18.18	15.5
	a04 (antiemetics and antinauseants)	434	30465	12.25	25.35
	a06 (drugs for constipation)	272	3870	7.68	3.22
	a07 (antidiarrhals, intestinal antiinflammatory/antiinfective agents)	195	4335	5.51	3.61
	a10 (drugs used in diabetes)	668	18653	18.86	15.52
	a11 (vitamins)	277	6824	7.82	5.68
	a12 (mineral supplements)	196	4458	5.53	3.71
	b03 (antianemic preparations)	228	4964	6.44	4.13
	b05 (blood substitutes and perfusion solutions)	1898	50618	53.59	42.12
	c01 (cardiac therapy)	382	10147	10.78	8.44
	c02 (antihypertensives)	194	4886	5.48	4.07
	c03 (diuretics)	1206	22680	34.05	18.87
	c07 (beta blocking agents)	914	24932	25.8	20.74
	c09 (agents acting on the renin-angiotensin system)	514	20193	14.51	16.8
	c10 (lipid modifying agents)	428	19228	12.08	16
	d01 (antifungals for dermatological use)	351	3588	9.91	2.99
	g04 (urologicals)	232	6294	6.55	5.24
	h02 (corticosteroids for systemic use)	458	10582	12.93	8.8
	h03 (thyroid therapy)	300	8510	8.47	7.08
	j01 (antibacterials for systemic use)	1104	23709	31.17	19.73
	l01 (antineoplastic agents)	202	4091	5.7	3.4
	m01 (antiinflammatory and antirheumatic products)	19	9944	0.54	8.27
	n02 (analgesics)	2370	85217	66.91	70.9
	n03 (antiepileptics)	405	9413	11.43	7.83
	n05 (psycholeptics)	1491	45239	42.09	37.64
	n06 (psychoanaleptics)	520	14490	14.68	12.06
	p01 (antiprotozoals)	189	3144	5.34	2.62
	r03 (drugs for obstructive airway diseases)	447	9138	12.62	7.6
Zip Code	urban	1983	64588	55.99	53.74
	semi-rural	1028	32172	29.02	26.77

**Modes of admission: "After consultation with doctor from the establishment" is when a patient is seen in a scheduled consultation and kept in hospital immediately afterwards, without going through the emergency room, therefore without immediate vital risk; "from home" is when the patient is transferred from their home to hospital by ambulance. ICU : intensive care unit

Table S3 (b). Number of medications in the observation time frame

	min	max	sd	mean	95% CI *
**case**	0	19	± 3.87	5.75	5.61-5.87
**control**	0	24	± 3.31	5.01	4.99-5.02

*p-value < 0.0001

Table S4 (a) Correlation analysis categorical variables (Pearson correlation coefficient >0.9)

New (variable used in model)	Variable	Correlation coefficient
Plasma ALT (GPT) [UI/L] - tested	Plasmatic GGT (Gamma glutamyl transferase) [UI/L] - tested	1.00
	plasmatic GOT (AST) [UI/L] - tested	1.00
Plasmatic uric acid [mmol/l] - tested	Bicarbonates - total CO2 [mmol/l] - tested	1.00
	Bicarbonates [mmol/l] - tested	1.00
	conjugated bilirubin [µmol/l] - tested	0.94
	total bilirubin [µmol/l] - tested	0.94
	Calcium [mmol/l] -tested	1.00
	Chlorides [mmol/l] -tested	0.90
	Creatinine [µmol/l] -tested	0.94
	Base excess [mmol/l] - tested	1.00
	Glycemia [mmol/l] - tested	1.00
	Lactate [mmol/l] - tested	1.00
	Phosphorus [mmol/l] - tested	1.00
	Plasmatic potassium (K) [mmol/l] - tested	1.00
	Sodium (Na) [mmol/l] - tested	1.00
	Urea [mmol/l] -tested	1.00
Albumin [g/l] - tested	Basophils [G/l] - tested	0.98
	mean corpuscular hemoglobin concentration (MCHC) [g/l] - tested	1.00
	Erythrocytes [T/l] - tested	0.98
	Eosino # [G/l] - tested	0.98
	FACTOR II [%] - tested	0.95
	FACTOR X [%] - tested	0.95
	Factor V [%] - tested	0.95
	Hematocrit [l/l] - tested	0.98
	Hb (measured) [g/l] - tested	1.00
	Red blood cell distribution width (RDW)[%] - tested	0.95
	Leucocytes [G/l] - tested	0.98
	Lympho # [G/l] - tested	0.98
	Mono # [G/l] - tested	0.98
	Neutro # [G/l] - tested	0.98
	Reticulocytes [G/l] - tested	0.98
	Prothrombin rate [%] - tested	0.95
	MCHC [pg] - tested	0.98
	Thrombocytes [G/l] - tested	0.98
	mean corpuscular volume (MCV) [fl] - tested	0.98
Plasmatic CPK [UI/l] - tested	Plasmatic lipase [UI/l] - tested	1.00
Blood O2 content [vol%] - tested	pCO2 [kPa] - tested	0.99
	pO2 [kPa] - tested	0.99
CRP (C-reactive protein) [mg/l] - tested	Prealbumin [mg/l] - tested	1.00
Bicarbonates [mmol/l] - normal	plasmatic protein [g/l] - tested	0.93
Plasmatic lactate dehydrogenase (LDH) [UI/l] - tested	Plasmatic alkaline phosphatase [UI/l] - tested	1.00

Table S4 (b). Correlation analysis continuous variables for SDOH (Pearson correlation coefficient >0.9)

new (variable used in model)	variable	correlation coefficient
C18_MENFAM	C18_MENCOUPAENF	0.90
P18_RP_5PP	P18_RP_VOIT2P	0.92
P18_CHOM1564	P18_CHOM2554	0.97

See table S1 for code description

Table S4 (c). Categories for correlated laboratory tests (Pearson correlation coefficient >0.9)

Laboratory test	Category
Hb (measured) [g/l] - tested	complete blood count
Hematocrit [l/l] - tested	
Erythrocytes [T/l] - tested	
mean corpuscular hemoglobin concentration [g/l] - tested	
mean cell hemoglobin [pg] - tested	
mean corpuscular volume [fl] - tested	
Leucocytes [G/l] - tested	
Neutro # [G/l] - tested	
Mono # [G/l] - tested	
Eosino # [G/l] - tested	
Basophils [G/l] - tested	
Lympho # [G/l] - tested	
Thrombocytes [G/l] - tested	
Reticulocytes [G/l] - tested	Reticulocytes
Plasmatic potassium (K) [mmol/l] - tested	electrolytes
Sodium (NA) [mmol/l] - tested	
Calcium [mmol/l] -tested	
Chlorides [mmol/l] -tested	
Phosphorus [mmol/l] - tested	
Bicarbonates - total CO2 [mmol/l] - tested	Blood gas test
Bicarbonates [mmol/l] - tested	
pCO2 [kPa] - tested	
pO2 [kPa] - tested	
Base excess [mmol/l] - tested	
Blood O2 content [vol%]	Oxygen saturation
Plasma ALT (GPT) [UI/L] - tested	liver enzymes
Plasmatic GGT (Gamma glutamyl transferase) [UI/L] -tested	
Plasmatic GOT (AST) [UI/L] - tested	
FACTOR II [%] - tested	Coagulation tests
FACTOR X [%] - tested	
Factor V [%] - tested	
Prothrombin rate [%] - tested	
CRP (C-reactive protein) [mg/l] - tested	CRP
Plasmatic uric acid [mmol/l] - tested	Routine Kidney Function Blood Test
Creatinine [µmol/l] -tested	
Urea [mmol/l] -tested	
conjugated bilirubin [µmol/l] - tested	Bilirubin

Laboratory test	Category
Albumin [g/l] - tested	Albumin
Plasmatic alkaline phosphatase [Ul/l] - tested	Alkaline Phosphatase
Glycemia [mmol/l] - tested	Blood Glucose
Lactate [mmol/l] - tested	Lactate
Plasmatic lactate dehydrogenase (LDH) [Ul/l] - tested	LDH
Plasmatic CPK [UI/l] - tested	CPK
Plasmatic lipase [UI/l] - tested	Lipase
Prealbumin [mg/l] - tested	Prealbumin
plasmatic protein [g/l] - tested	Blood protein

Table S5 (a). All primary diagnoses from cases and controls during their hospitalization with a false discovery rate (FDR) of <0.01, ordered by odds ratio

Primary Diagnosis	odds ratio (95% CI)	FDR	n case	% case	n control	% control
R57-Cardiogenic shock	12.51 (10.94-14.32)	<0.0001	304	8.58	895	0.74
C34-Malignant neoplasma of main bronchus	12.08 (10.32-14.14)	<0.0001	217	6.13	646	0.54
J96-Acute respiratory failure	9.06 (7.90-10.38)	<0.0001	276	7.79	1111	0.92
I50-Heart failure	3.45 (3.06-3.90)	<0.0001	310	8.75	3250	2.7
Z51-Admission for antineoplastic radiation therapy	3.09 (2.73-3.49)	<0.0001	303	8.55	3531	2.94
I63-Cerebral infarction due to thrombosis of precerebral arteries	2.90 (2.49-3.37)	<0.0001	188	5.31	2282	1.9

Table S5 (b). All secondary diagnoses from cases and controls during their hospitalization with a false discovery rate (FDR) of <0.01, ordered by odds ratio

Secondary Diagnosis	odds ratio (95% CI)	False discovery rate	n case	% case	n control	% control
I46-Cardiac arrest	51.36 (43.50-60.64)	<0.0001	345	9.74	252	0.21
R57-Cardiogenic shock	20.22 (18.30-22.33)	<0.0001	670	18.92	1371	1.14
K72-Acute and subacute hepatic failure	18.88 (16.61-21.46)	<0.0001	379	10.7	758	0.63
R40-Somnolence	15.02 (13.92-16.21)	<0.0001	1216	34.33	4042	3.36
R34-Anuria and oliguria	11.11 (9.96-12.39)	<0.0001	467	13.18	1621	1.35
N17-Acute kidney failure with tubular necrosis	10.45 (9.52-11.48)	<0.0001	655	18.49	2553	2.12
J69-Pneumonitis due to inhalation of food and vomit	9.23 (8.14-10.48)	<0.0001	326	9.2	1305	1.09
J96-Acute respiratory failure	8.41 (7.77-9.10)	<0.0001	959	27.08	5082	4.23
C78-Secondary malignant neoplasm of lung	8.39 (7.65-9.20)	<0.0001	647	18.27	3119	2.6
C79-Secondary malignant neoplasm of kidney and renal pelvis	8.21 (7.40-9.10)	<0.0001	500	14.12	2360	1.96
J91-Malignant pleural effusion	7.46 (6.49-8.56)	<0.0001	258	7.28	1253	1.04
G93-Cerebral cysts	7.15 (6.37-8.02)	<0.0001	378	10.67	1976	1.64
Z49-Preparatory care for renal dialysis	7.05 (6.20-8.02)	<0.0001	301	8.5	1562	1.3
Z51-Admission for antineoplastic radiation therapy	6.55 (6.04-7.11)	<0.0001	854	24.11	5557	4.62
R18-Malignant ascites	6.50 (5.71-7.40)	<0.0001	291	8.22	1633	1.36
K92-Hematemesis	6.06 (5.21-7.05)	<0.0001	207	5.84	1219	1.01
C77-Secondary and unspecified malignant neoplasm of lymph nodes of head, face and neck	5.73 (5.07-6.48)	<0.0001	320	9.03	2047	1.7
J15-Pneumonia due to Klebsiella pneumoniae	5.48 (4.84-6.20)	<0.0001	314	8.87	2096	1.74
J18-Bronchopneumonia, unspecified organism	5.38 (4.59-6.29)	<0.0001	188	5.31	1240	1.03
R53-Neoplastic (malignant) related fatigue	4.91 (4.53-5.31)	<0.0001	877	24.76	7554	6.29
D63-Anemia in neoplastic disease	4.82 (4.17-5.58)	<0.0001	217	6.13	1604	1.33
J98-Diseases of bronchus, not elsewhere classified	4.68 (4.09-5.36)	<0.0001	254	7.17	1952	1.62
I50-Heart failure	4.64 (4.28-5.04)	<0.0001	797	22.5	7071	5.88
E43-Unspecified severe protein-calorie malnutrition	4.49 (4.10-4.91)	<0.0001	633	17.87	5559	4.63
C34-Malignant neoplasm of main bronchus	4.44 (3.85-5.13)	<0.0001	221	6.24	1773	1.48
K56-Paralytic ileus	4.44 (3.86-5.11)	<0.0001	233	6.58	1875	1.56
G81-Flaccid hemiplegia	4.35 (3.81-4.97)	<0.0001	261	7.37	2159	1.8
A41-Sepsis due to Staphylococcus aureus	4.35 (3.88-4.87)	<0.0001	369	10.42	3131	2.61
L89-Pressure ulcer of elbow	4.34 (3.93-4.80)	<0.0001	489	13.81	4275	3.56
E86-Dehydration	4.24 (3.81-4.72)	<0.0001	417	11.77	3665	3.05
E87-Hyperosmolality and hypernatremia	4.23 (3.95-4.54)	<0.0001	1348	38.06	15235	12.68
R39-Extravasation of urine	3.94 (3.59-4.33)	<0.0001	565	15.95	5522	4.59
R06-Dyspnea	3.78 (3.43-4.15)	<0.0001	537	15.16	5432	4.52
R52-Pain, unspecified	3.67 (3.35-4.03)	<0.0001	571	16.12	5977	4.97
R13-Aphagia	3.61 (3.20-4.07)	<0.0001	315	8.89	3164	2.63
D69-Allergic purpura	3.49 (3.02-4.03)	<0.0001	213	6.01	2165	1.8

Secondary Diagnosis	odds ratio (95% CI)	False discovery rate	n case	% case	n control	% control
R41-Disorientation, unspecified	3.43 (3.14-3.75)	<0.0001	622	17.56	7026	5.85
B37-Candidal stomatitis	3.25 (2.87-3.68)	<0.0001	292	8.24	3233	2.69
R60-Localized edema	3.18 (2.87-3.51)	<0.0001	477	13.47	5613	4.67
F05-Delirium due to known physiological condition	3.09 (2.69-3.55)	<0.0001	231	6.52	2654	2.21
R45-Nervousness	3.02 (2.70-3.39)	<0.0001	349	9.85	4192	3.49
E83-Disorders of copper metabolism	2.98 (2.56-3.46)	<0.0001	190	5.36	2247	1.87
I48-Paroxysmal atrial fibrillation	2.92 (2.70-3.15)	<0.0001	971	27.41	13774	11.46
R65-Symptoms and signs specifically associated with systemic inflammation and infection	2.90 (2.67-3.15)	<0.0001	750	21.17	10179	8.47
J90-Pleural effusion, not elsewhere classified	2.88 (2.54-3.28)	<0.0001	271	7.65	3357	2.79
K76-Fatty (change of) liver, not elsewhere classified	2.87 (2.49-3.31)	<0.0001	222	6.27	2735	2.28
K83-Cholangitis	2.74 (2.36-3.19)	<0.0001	191	5.39	2445	2.03
R47-Dysphasia and aphasia	2.63 (2.32-2.97)	<0.0001	291	8.22	3960	3.29
F41-Panic disorder [episodic paroxysmal anxiety]	2.50 (2.24-2.79)	<0.0001	382	10.78	5541	4.61
E46-Unspecified protein-calorie malnutrition	2.40 (2.18-2.64)	<0.0001	516	14.57	7976	6.64
R63-Anorexia	2.35 (2.07-2.67)	<0.0001	272	7.68	4111	3.42
R74-Nonspecific elevation of levels of transaminase and lactic acid dehydrogenase [LDH]	2.34 (2.01-2.73)	<0.0001	187	5.28	2795	2.33
Z74-Reduced mobility	2.31 (2.05-2.59)	<0.0001	330	9.32	5127	4.27
N18-Chronic kidney disease (CKD)	2.27 (2.08-2.48)	<0.0001	640	18.07	10643	8.86
J44-Chronic obstructive pulmonary disease with acute lower respiratory infection	2.17 (1.95-2.42)	<0.0001	382	10.78	6336	5.27
I70-Atherosclerosis of aorta	2.00 (1.78-2.24)	<0.0001	332	9.37	5919	4.92
R33-Drug induced retention of urine	1.98 (1.74-2.25)	<0.0001	262	7.4	4660	3.88
F06-Psychotic disorder with hallucinations due to known physiological condition	1.94 (1.69-2.23)	<0.0001	228	6.44	4109	3.42
N39-Urinary tract infection, site not specified	1.92 (1.65-2.23)	<0.0001	188	5.31	3417	2.84
I49-Ventricular fibrillation and flutter	1.89 (1.62-2.21)	<0.0001	180	5.08	3308	2.75
R10-Acute abdomen	1.79 (1.55-2.06)	<0.0001	209	5.9	4074	3.39
I35-Nonrheumatic aortic (valve) stenosis	1.78 (1.53-2.07)	<0.0001	181	5.11	3532	2.94
B96-Mycoplasma pneumoniae [M. pneumoniae] as the cause of diseases classified elsewhere	1.77 (1.59-1.96)	<0.0001	421	11.89	8513	7.08
I25-Chronic ischemic heart disease	1.74 (1.58-1.91)	<0.0001	537	15.16	11194	9.31
R50-Fever of other and unknown origin	1.66 (1.47-1.87)	<0.0001	305	8.61	6456	5.37
I34-Nonrheumatic mitral (valve) insufficiency	1.57 (1.36-1.81)	<0.0001	208	5.87	4599	3.83
Z95-Presence of cardiac pacemaker	1.56 (1.44-1.70)	<0.0001	704	19.88	16463	13.7
R00-Tachycardia, unspecified	1.54 (1.34-1.78)	<0.0001	212	5.99	4758	3.96
R11-Nausea	1.46 (1.29-1.66)	<0.0001	273	7.71	6491	5.4
E11-Type 2 diabetes mellitus with hyperosmolarity	1.46 (1.34-1.59)	<0.0001	666	18.8	16484	13.72
B95-Streptococcus, group A, as the cause of diseases classified elsewhere	1.45 (1.27-1.65)	<0.0001	252	7.11	6035	5.02
I10-Essential (primary) hypertension	1.43 (1.34-1.53)	<0.0001	1467	41.42	39705	33.04
R26-Ataxic gait	1.41 (1.23-1.61)	<0.0001	247	6.97	6077	5.06
Z92-Personal history of contraception	1.40 (1.29-1.52)	<0.0001	778	21.96	20149	16.76
I44-Atrioventricular block, first degree	1.40 (1.20-1.63)	0,000158	179	5.05	4412	3.67
Z86-Personal history of in-situ and benign neoplasms and neoplasms of uncertain behavior	1.39 (1.24-1.55)	<0.0001	353	9.97	8878	7.39
E03-Congenital hypothyroidism with diffuse goiter	1.38 (1.19-1.60)	0,000125	197	5.56	4908	4.08
Z85-Personal history of malignant neoplasm of digestive organs	1.38 (1.21-1.56)	<0.0001	265	7.48	6671	5.55
Z99-Dependence on aspirator	1.37 (1.22-1.53)	<0.0001	344	9.71	8747	7.28
R70-Elevated erythrocyte sedimentation rate	1.36 (1.20-1.55)	<0.0001	265	7.48	6734	5.6
F10-Alcohol related disorders	1.33 (1.17-1.53)	0,000225	232	6.55	5996	4.99
F17-Nicotine dependence	0.81 (0.72-0.90)	0,000483	348	9.82	14308	11.9
D50-Iron deficiency anemia secondary to blood loss (chronic)	0.71 (0.61-0.83)	<0.0001	165	4.66	7717	6.42
E66-Obesity due to excess calories	0.46 (0.41-0.52)	<0.0001	310	8.75	20674	17.2
Z37-Single live birth	0.00 (0.00-0.03)	<0.0001	1	0.03	8258	6.87

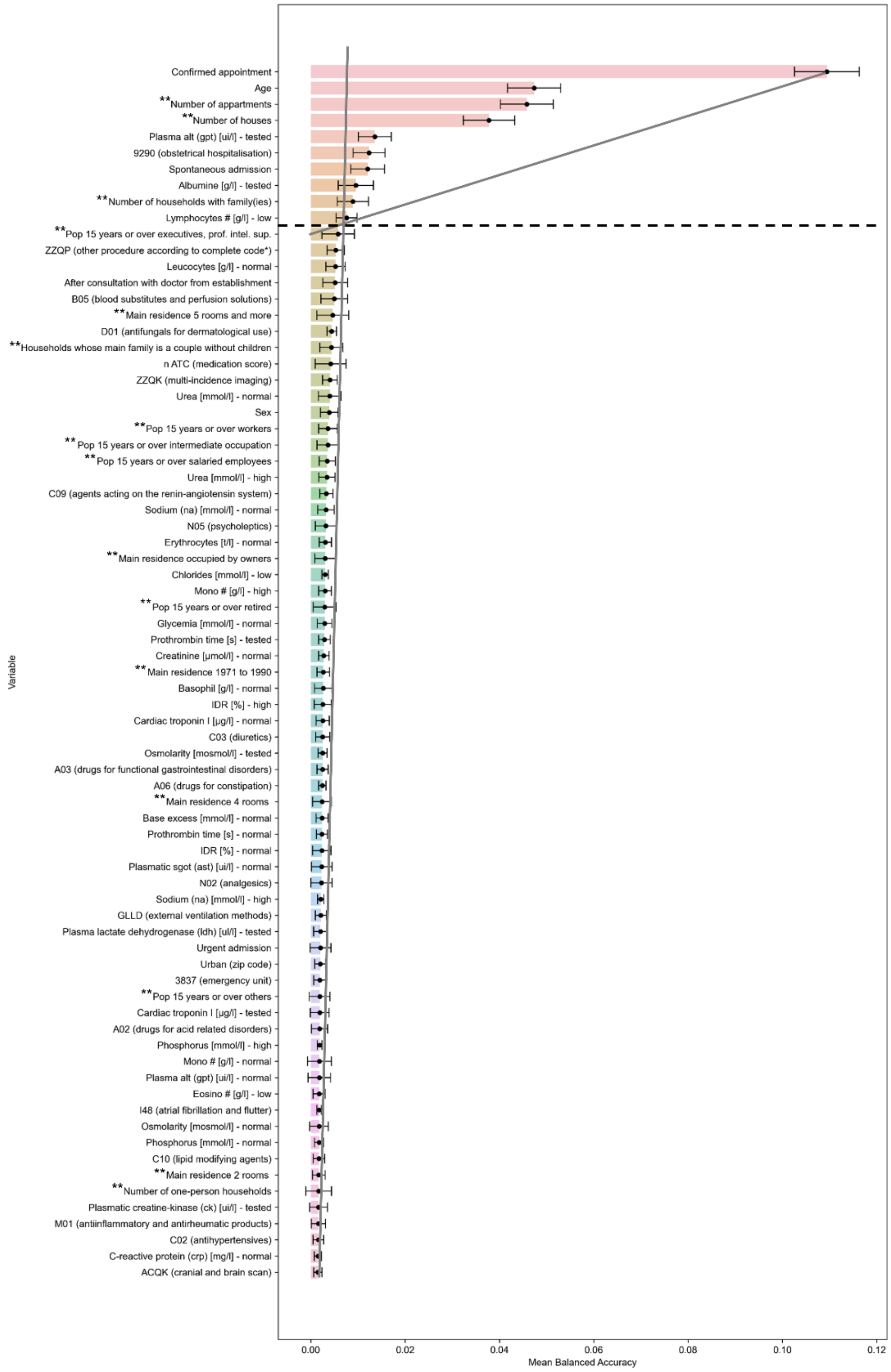

Figure S1. Top 75 most influential variables sorted by their contributing mean balanced accuracy for the support vector machine algorithm. Error bars indicate standard deviation based on 250 rounds of permutation importance. Dashed line shows the threshold used as a cut-off for the most important variables, based on the area where the slope of the curve flattens. ** social determinants of health.

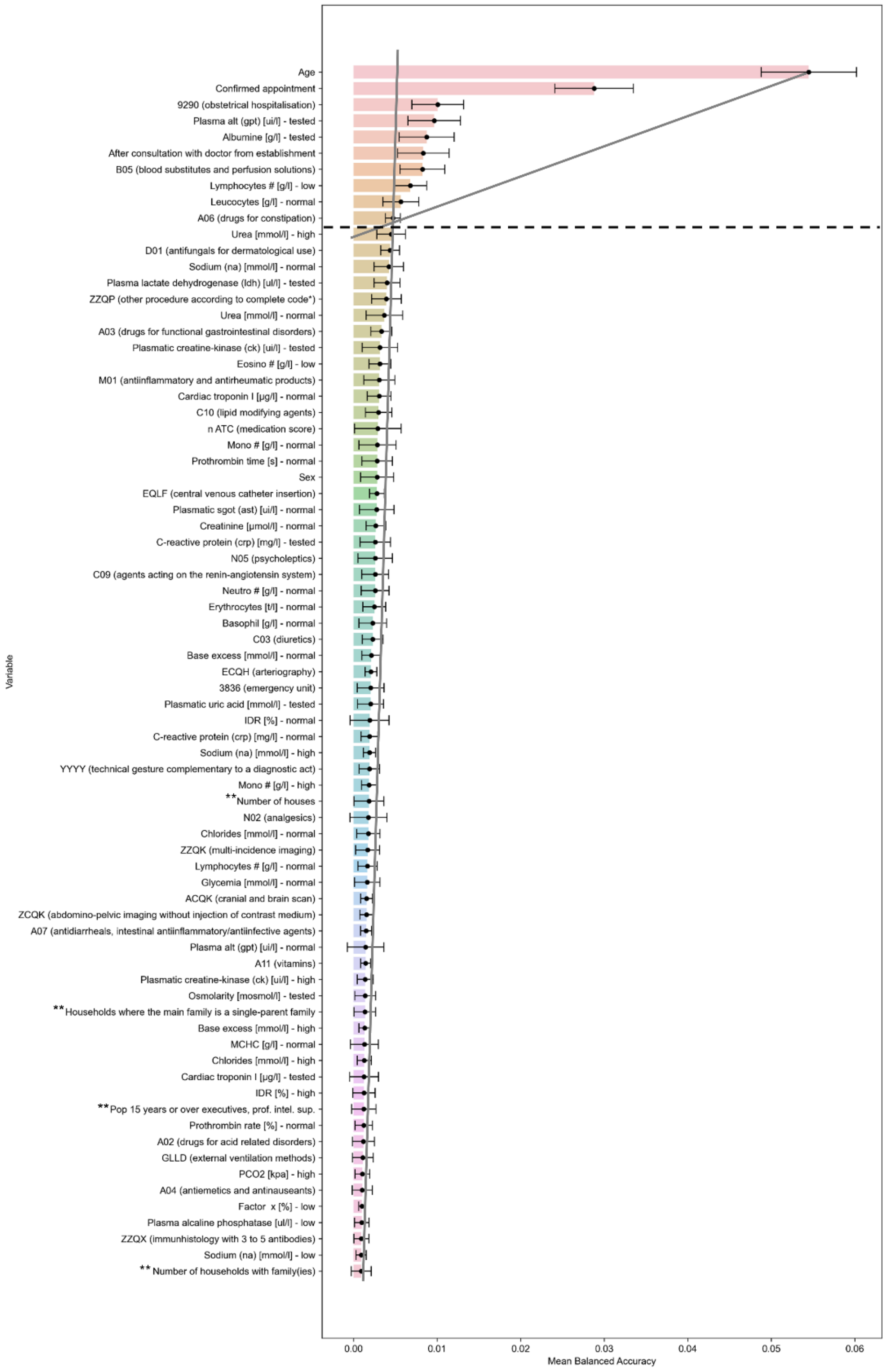

Figure S2. Top 75 most influential variables sorted by their contributing mean balanced accuracy for the logistic regression algorithm. Error bars indicate standard deviation based on 250 rounds of permutation importance. Dashed line shows the threshold used as a cut-off for the most important variables, based on the area where the slope of the curve flattens. ** social determinants of health.

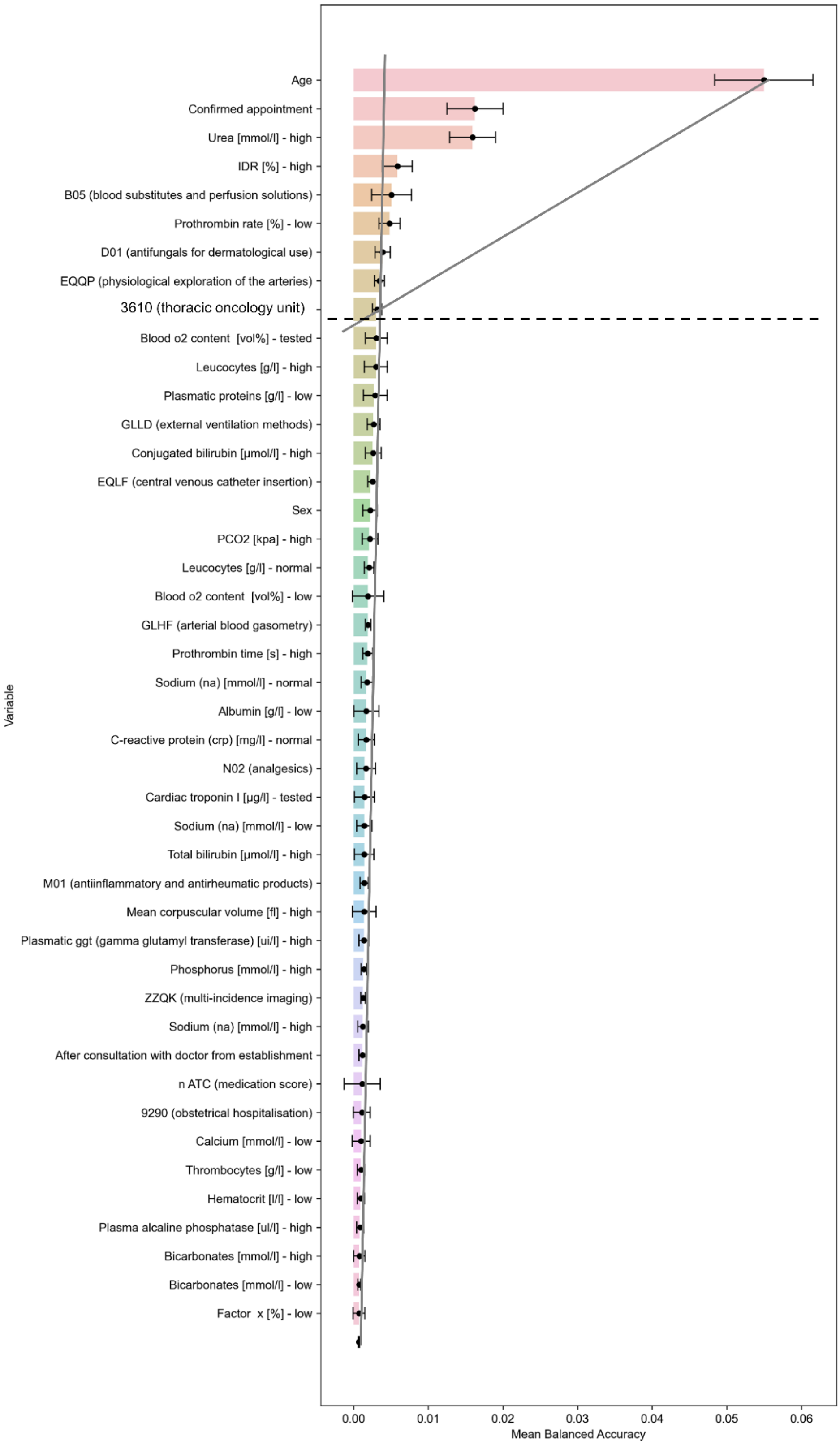

Figure S3. Top 45 most influential variables sorted by their contributing mean balanced accuracy for the xgboost algorithm. Error bars indicate standard deviation based on 250 rounds of permutation importance. Dashed line shows the threshold used as a cut-off for the most important variables, based on the area where the slope of the curve flattens. ** social determinants of health.

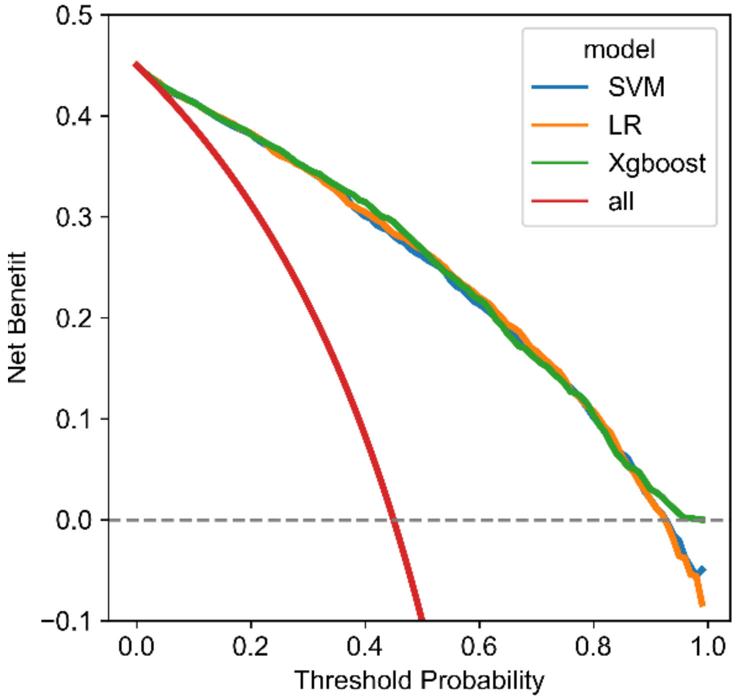

Figure S4. Decision curve analysis for the top three performing models using the full set of variables. Logistic regression (LR), support vector machine (SVM)
